# Sustaining stewardship: longitudinal evaluation of an integrated antimicrobial programme in the ICU

**DOI:** 10.1093/jac/dkag086

**Published:** 2026-03-17

**Authors:** Ashmitha Thomas, Sara Vogrin, Adele Batrouney, Misha Devchand, Sharmila Khumra, Shanti Narayanasamy, Satwik Motaganahalli, Jason A Trubiano, Stephen J Warrillow, Gemma K Reynolds

**Affiliations:** Department of Infectious Diseases & Immunology, Austin Health, Melbourne, Australia; Department of Infectious Diseases & Immunology, Austin Health, Melbourne, Australia; Department of Infectious Diseases & Immunology, Austin Health, Melbourne, Australia; Department of Pharmacy, Austin Health, Melbourne, Australia; Department of Infectious Diseases & Immunology, Austin Health, Melbourne, Australia; Department of Pharmacy, Austin Health, Melbourne, Australia; Department of Infectious Diseases & Immunology, Austin Health, Melbourne, Australia; Department of Pharmacy, Austin Health, Melbourne, Australia; Department of Infectious Diseases & Immunology, Austin Health, Melbourne, Australia; Department of Infectious Diseases, The Peter Doherty Institute for Infection and Immunity, The University of Melbourne, Melbourne, Australia; Department of Infectious Diseases & Immunology, Austin Health, Melbourne, Australia; Department of Infectious Diseases, The Peter Doherty Institute for Infection and Immunity, The University of Melbourne, Melbourne, Australia; Department of Infectious Diseases & Immunology, Austin Health, Melbourne, Australia; Department of Infectious Diseases, The Peter Doherty Institute for Infection and Immunity, The University of Melbourne, Melbourne, Australia; Department of Intensive Care, Austin Health, Melbourne, Australia; Department of Infectious Diseases & Immunology, Austin Health, Melbourne, Australia; Department of Infectious Diseases, The Peter Doherty Institute for Infection and Immunity, The University of Melbourne, Melbourne, Australia; National Centre for Infections in Cancer, Peter MacCallum Cancer Centre, Melbourne, Australia

## Abstract

**Objectives:**

To evaluate the long-term sustainability and impact of an integrated electronic medical record–driven antimicrobial stewardship (AMS) ward round in an ICU at a tertiary referral hospital. The study assessed antimicrobial prescribing patterns, acceptance of stewardship recommendations, and antimicrobial consumption over 7 years.

**Methods:**

A prospective review commenced with implementation of the ICU-AMS ward round at Austin Health in 2017. When AMS recommendations were given, data were collected including patient demographics, antimicrobial prescribing, classification of recommendation, and acceptance. Antimicrobial use was assessed via DDDs per occupied bed day per month and analysed using interrupted time series analysis. Logistic regression examined patient and clinician factors associated with recommendation acceptance.

**Results:**

Over 7 years, 9163 AMS recommendations were made for 4610 patients. Recommendation acceptance was high, with antibiotic escalation the most accepted (95%) and discontinuation least accepted (82%). Recommendations were more likely to be accepted in immunocompromised (OR 1.31, *P* = 0.003) and non-surgical patients (OR 1.31, *P* < 0.001). Recommendations provided by AMS physicians who identified as men were more likely to be accepted (OR 1.23, *P* = 0.003). Antimicrobial consumption trends showed significant decreases in piperacillin/tazobactam, meropenem, ciprofloxacin and vancomycin use post-implementation. Amoxicillin/clavulanate use increased, suggesting potential compensatory prescribing.

**Conclusions:**

This study demonstrates the long-term effectiveness and sustainability of an ICU-AMS programme, achieving high recommendation acceptance and sustained reductions in broad-spectrum antimicrobial use. Continued efforts should focus on optimizing stewardship practices, addressing barriers to acceptance, and evaluating compensatory prescribing patterns.

## Introduction

Antimicrobial stewardship (AMS) programmes in intensive care units (ICUs) reduce broad-spectrum antibiotic use, the incidence of MDR pathogens, antibiotic-associated adverse effects, and healthcare-associated costs.^1^ In-person delivery and feedback contribute to sustained improvements in antimicrobial consumption and cost;^2^ however, the long-term sustainability and impact of such programmes in the critically ill or ICU are infrequently examined.

## Methods

This study was approved by our institution’s Human Research Ethics Committee (HREC/98045).

Based on the principles of ‘Handshake Stewardship’, an ICU-AMS ward round was instituted at Austin Health in 2017 and remains a core AMS component at this 500-bed tertiary hospital. The model is defined by three key elements: (i) no restriction or preauthorization requirement, (ii) comprehensive review of all prescribed antimicrobials, and (iii) in-person feedback delivered by a pharmacist–physician team.^3^ As previously described, the ward round is conducted on weekdays and is integrated into the workload of the Infectious Diseases (ID) and ICU medical teams, and AMS pharmacist.^4^ The round involves asynchronous review via the electronic medical record (EMR) followed by in-person discussion in the ICU. All patients receiving antimicrobials, or for whom antimicrobials are being considered, are discussed. Whereas pharmacist input is integral to case review and medication optimization, stewardship recommendations are communicated by the ID clinician during the round. Recommendations are classified according to the internally developed ‘5 Moments of Antimicrobial Prescribing’: escalation, de-escalation, discontinuation, oral switch and optimization. This metric was created by internal consensus to provide a framework to assess AMS compliance.^4^

An initial evaluation conducted in the first 6 months of implementation assessed recommendation acceptance, target antibiotic use and prescribing appropriateness, with follow-up at 2 years reporting sustained changes in antimicrobial use and further improvements in appropriateness.^4,5^ Building on these early evaluations, we conducted a prospective study of the 7 year sustainability and impact of the integrated AMS service from August 2017 to December 2024. We also examined patient- and clinician-level social factors that may influence the uptake of ‘handshake stewardship’.

The primary outcome was antimicrobial consumption measured through the National Antimicrobial Utilisation Surveillance Program (NAUSP)—a volume-based method for monitoring and benchmarking use of antimicrobials between Australian hospitals, expressed as DDDs per 1000 occupied bed days (OBDs), calculated monthly.^6^ Institutional standard dosing regimens for target antimicrobials are summarized in Table S1 (available as Supplementary data at *JAC* Online). Changes in antimicrobial consumption over time were analysed using interrupted time series analysis using ordinary least squares regression with Newey–West standard errors.

Secondary outcomes included AMS recommendation type, classified by the ‘5 Moments’ framework,^4^ and acceptance by the ICU team within 24 h. Patient demographics and admitting unit were recorded. Immunocompromised patients were identified by admitting unit (liver and renal transplant, haematology, oncology, rheumatology). The relationship between acceptance of recommendation, and patient/clinician characteristics was analysed using univariable logistic regression, overall and separately for each type of recommendation. Standard errors were adjusted for clustering within the participant. Results are expressed as ORs with 95% CIs. All analyses were performed using Stata 18 (StataCorp LLC).

## Results

The ICU-AMS service provided recommendations for 4610 patients during the study period. Median age was 62 years (IQR 50–73), with 60.2% male predominance (Table S2). Most patients were admitted under medical units (63.4%), and 835 (18.1%) were immunocompromised. Over a 7 year period (2017–2024) there were 1078 rounds, with an average duration of 30 min (IQR 25–40). The most reviewed antibiotics were piperacillin/tazobactam, ceftriaxone and vancomycin (1906, 1162 and 766 instances, respectively).

**Figure 1.**
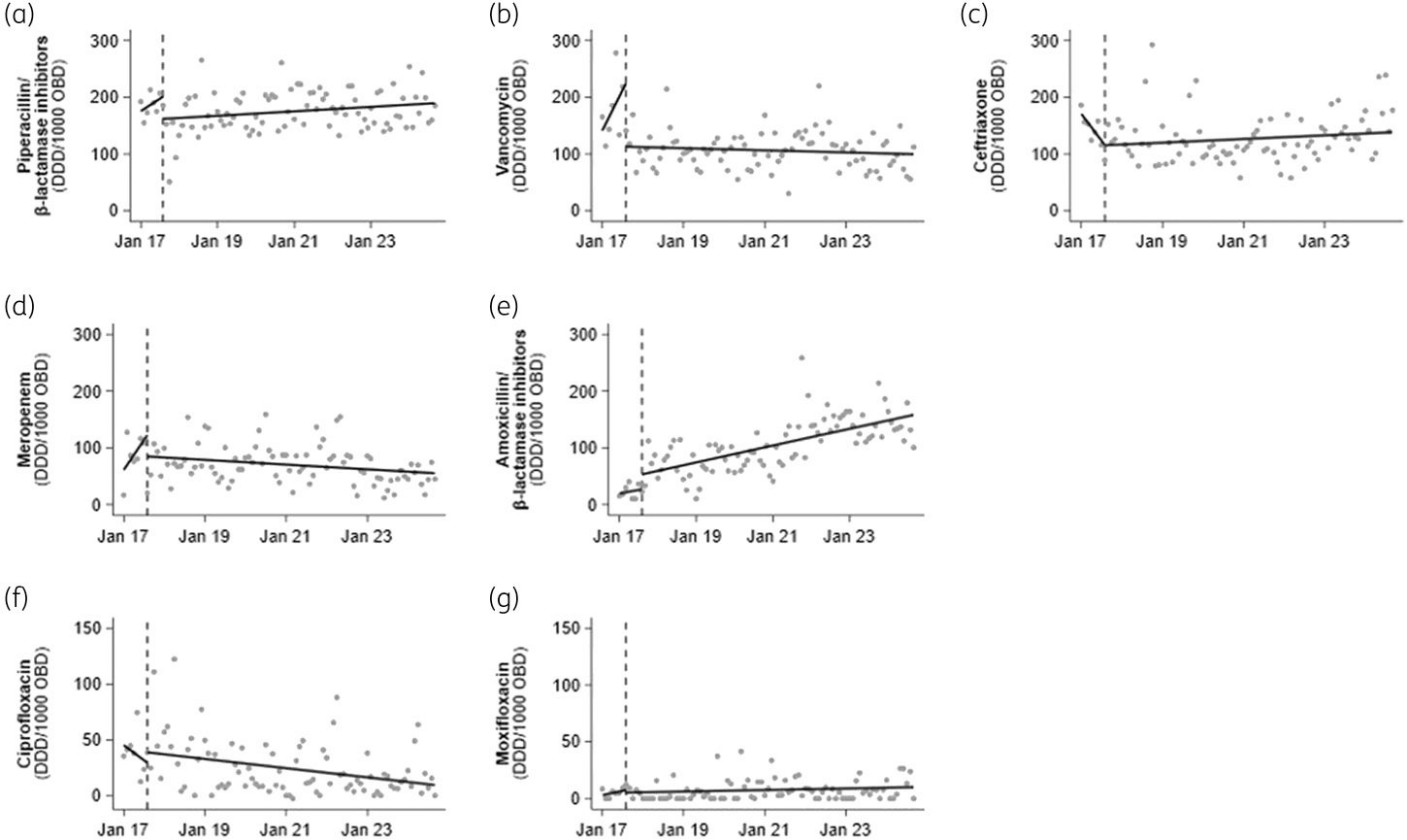
DDD antibiotic use for commonly targeted antibiotics in the ICU 6 months before and 7 years after ICU-AMS intervention. Dotted vertical lines represent start of intervention (August 2017). Solid lines represent pre- and post-intervention trends in antimicrobial use estimated using interrupted time series analysis. The dots on the graph are raw data points. (a) piperacillin/tazobactam, (b) vancomycin, (c) ceftriaxone, (d) meropenem, (e) amoxicillin/clavulanate, (f) ciprofloxacin, (g) moxifloxacin.

Antimicrobial consumption of target agents is shown in Figure 1. Additional consumption data are found in Table S3 and Figures S1 and S2. Prior to programme implementation in 2017, piperacillin/tazobactam use was rising (3.6 DDD/1000 OBD/month; 95% CI, 1.3–5.9; *P* = 0.003); post-intervention it decreased (−38.9 DDD/1000 OBD/month; 95% CI, −60 to 17.4; *P* = 0.001) without significant change in trend thereafter (0.32 DDD/1000 OBD/month; 95% CI, −0.01 to 0.7; *P* = 0.059). Meropenem use also decreased at implementation (−37.7 DDD/1000 OBD/month; 95% CI, −66.1 to 9.4; *P* = 0.01), with continued decline over subsequent years (−0.3 DDD/1000 OBD/month; 95% CI, −0.6 to 0.1; *P* = 0.021). Vancomycin use decreased immediately after programme commencement (−111.7 DDD/1000 OBD/month; 95% CI, −193.2 to −30.2; *P* = 0.008), with no significant change since (−0.2 DDD/1000 OBD/month; 95% CI, −0.4 to 0.1; *P* = 0.277). Ciprofloxacin showed a sustained reduction (−0.35 DDD/1000 OBD/month; 95% CI, −0.6 to −0.1; *P* = 0.004) with both oral and IV formulations showing decline (Figure S1); moxifloxacin use remained low and unchanged (0.06 DDD/1000 OBD/month; 95% CI, −0.0 to 0.1; *P* = 0.124). No significant change was seen in ceftriaxone use. Amoxicillin/clavulanate in both oral and IV formulations increased post-intervention (26.5 DDD/1000 OBD/month; 95% CI, 6.9–46.0; *P* = 0.009), with continued rise long-term (1.2 DDD/1000 OBD/month; 95% CI, 0.9-1.6; *P* < 0.001), although the upward trend had plateaued (Figure S2).

A total of 9163 recommendations were made over 7 years, with overall acceptance of 84.9% (Table 1 and Figures S3 and S4). Discontinuation was the most common recommendation (43.2%) but had the lowest overall acceptance rate (82.3%). Factors associated with acceptance of recommendations within 24 h are presented in Table S4. Recommendations for immunocompromised patients were more likely to be accepted overall (OR 1.31; 95% CI, 1.09–1.57; *P* = 0.003), and for antibiotic discontinuation (OR 1.33; 95% CI, 1.04–1.70; *P* = 0.024). Recommendations made for non-surgical patients were also more likely to be accepted (OR 1.31; 95% CI, 1.14–1.5; *P* < 0.001). Targeted sub-analysis demonstrated de-escalation and discontinuation recommendations (Table S5) were more likely to be accepted for meropenem compared with other antibiotics (87.7% versus 82.6%, *P* = 0.009).

**Table 1.** Recommendations and acceptance within 24 h of each of the ‘5 Moments of Antimicrobial Prescribing’

AMS moments	Recommendations, *n* (%)	Accepted, *n* (%)
Escalation	873 (9.5)	827 (94.7)
De-escalation	1500 (16.4)	1300 (86.7)
Discontinuation	3958 (43.2)	3257 (82.3)
Switch	951 (10.4)	793 (83.4)
Optimization	1881 (20.5)	1604 (85.3)

*Escalation:* broaden the spectrum of activity based on guidelines or microbiology test results. *De-escalation:* narrow the spectrum of activity based on guidelines or microbiology test results. *Discontinuation:* cease an antimicrobial due to unlikely infection, recommended duration of therapy reached, or unnecessary spectrum of antimicrobial activity. *Switch:* switch from IV to oral antimicrobials, change to alternative IV antimicrobial with similar spectrum of activity. *Optimization:* enter a cease date in the electronic medical record, modify the dose regimen based on patient- or infection-related factors, manage drug–drug interactions, additional microbiology tests or therapeutic drug monitoring required.

Psychosocial factors including round duration, ID physician gender, and seniority were also analysed (Table S4). In univariable analysis, overall recommendations by ID clinicians who identified as men were more likely to be accepted (OR 1.23; 95% CI, 1.07–1.4; *P* = 0.003). This association was further examined for effect of seniority, using a gender-by-seniority interaction, which was not significant, suggesting that effect of gender was consistent across seniority strata. ID clinician seniority was associated with increased acceptance of de-escalation recommendations (OR 1.03; 95% CI, 1.01–1.05; *P* = 0.01). Longer rounds (per 10 min increment) were associated with higher rates of acceptance (OR 1.10; 95% CI, 1.02–1.19; *P* = 0.018), particularly for antibiotic discontinuation (*P* = 0.007) and oral switch (*P* = 0.016).

## Discussion

This long-term evaluation found that a structured, integrated AMS programme was sustainable and effective, with high recommendation acceptance and reduced broad-spectrum antimicrobial use. Escalation had the highest acceptance, whereas discontinuation had the lowest, consistent with prior data in our cohorts and others,^5–7^ likely reflecting the high acuity and diagnostic uncertainty common in ICU settings, where clinicians may be more reluctant to withdraw antimicrobials due to risk of deterioration. However, the overall good compliance with discontinuation recommendations (82%), coupled with an association between increased acceptance and longer clinical discussion, reinforces the value of direct clinician engagement compared with other stewardship strategies.^8^ Notably, de-escalation or discontinuation recommendations involving meropenem were accepted more frequently than for other agents, which may reflect clearer microbiology-driven triggers for narrowing therapy once a carbapenem has been started, or greater clinician prioritization of carbapenem-sparing stewardship. Although this study did not directly assess the effect of an AMS programme on patient outcomes, it is reassuring to note that survival for patients with infective diagnoses and sepsis remained consistent over the study period and compares favourably with peer ICUs according to independent external benchmarking (Figures S5–S7).

The shift in antimicrobial use highlights the programme’s impact. We demonstrated successful influence on prescribing practices, leading to sustained reduction in use of meropenem, piperacillin/tazobactam, vancomycin and ciprofloxacin, despite representing a tertiary ICU servicing solid-organ transplant and haematology-oncology patients. The observed increase in amoxicillin/clavulanate use may indicate compensatory prescribing trends. Although this may reflect improved de-escalation or promotion of oral switch, further evaluation is needed to confirm alignment with AMS goals.

Generalizability of this model to ICUs with a higher antimicrobial resistance burden, or in less-resourced settings may vary. Although the core ‘handshake stewardship’ principles are transferable, the magnitude and pattern of change in consumption may differ where baseline broad-spectrum use is higher and de-escalation options are constrained by local resistance epidemiology.^9^ In such settings, stewardship impact may depend more heavily on rapid diagnostics, local antibiograms and availability of effective carbapenem-sparing regimens. Implementation in many low- and middle-income country (LMIC) contexts may also be further limited because ‘lift-and-shift’ stewardship models are reliant on local health system capacity for adaptation.^10^

Acceptance of AMS recommendations appears to be shaped by patient-specific, clinical and social factors. Higher acceptance in immunocompromised patients may be evidence of increased receptiveness to specialist input in complex cases. This finding in our cohort aligns with growing evidence supporting AMS effectiveness in this population. Other studies have also demonstrated that AMS interventions in high-risk patients, such as those with haematological malignancies or undergoing chemotherapy, can safely reduce antimicrobial exposure without adverse outcomes, and may even be associated with improved survival.^11,12^ The reduced acceptance of AMS recommendations among non-medical patients in our study reflects well-documented challenges in surgical populations.^13,14^ AMS efforts have traditionally focused on perioperative prophylaxis, with less emphasis on postoperative therapeutic prescribing.^15^ Adherence to antibiotic guidelines is often inconsistent, influenced by individual clinician preferences, institutional protocols, and concerns about postoperative infections,^13^ where diagnostic uncertainty can foster reluctance to de-escalate antimicrobials.

The observed association between ID clinician gender and recommendation acceptance suggests that interpersonal dynamics and team structures may play a role; however, confounding related to clinician experience, case-mix and temporal changes remains possible. Recent survey and interviews of stewardship providers found that those who identified as women were almost three times more likely to report experience of bias, compared with counterparts who identified as men.^16^ These findings highlight the need for qualitative research and interventions co-designed with the relevant stakeholders, to help clarify these social determinants and support consistent uptake of stewardship advice across varied clinical settings; sustainable AMS programmes must attend not only to prescribing behaviours and outcomes, but also to the interpersonal and organizational context in which they operate.

This study has some limitations. The ICU-AMS service exists within a strong secular culture of AMS within our institution, with pre-approval prescribing restrictions,^4,5^ other sub-specialty handshake models,^17^ and a comprehensive antibiotic allergy service.^18^ Nonetheless, our programme shows enduring changes in a high-acuity unit, with increased patient complexity, which has unique pressures toward frequent and broader-spectrum prescribing.^19^ Reasons for non-acceptance were not systematically captured across the study period as such barriers to uptake are not explored. Patient-level outcomes such as length of stay, mortality or re-admission were not assessed. Antibiotic consumption, although standardized, may be influenced by institutional dosing practices (e.g. augmented renal clearance, renal replacement therapy, and extended-infusion protocols). Finally, generalizability may vary, particularly in lower-resource settings, and in high antimicrobial resistance settings where prescribing patterns and de-escalation options are shaped by local antibiograms and formulary constraints.

This study underscores the long-term viability of an integrated AMS service in a critical care setting. Sustained reduction in broad-spectrum antibiotic use, high recommendation acceptance rates, and ongoing engagement over several years highlight the value of this ICU-AMS programme. These findings, based on a uniquely long-term study in this setting, support the role of AMS in reducing unnecessary antimicrobial exposure, in turn decreasing the selection pressure for MDR organisms, and ultimately improving patient outcomes.^20^ Future efforts should focus on maintaining and enhancing these gains, and explore the qualitative drivers of AMS acceptance, to develop a more nuanced and tailored ‘handshake’.

## Supplementary Material

dkag086_Supplementary_Data

## Supplementary data

Figures S1 to S7 and Tables S1 to S5 are available as Supplementary data at *JAC* Online.

## References

[1] Timsit J-F, Bassetti M, Cremer O et al Rationalizing antimicrobial therapy in the ICU: a narrative review. Intensive Care Med 2019; 45: 172–89. 10.1007/s00134-019-05520-5 30659311

[2] Morris AM, Bai A, Burry L et al Long-term effects of phased implementation of antimicrobial stewardship in academic ICUs: 2007-2015. Crit Care Med 2019; 47: 159–66. 10.1097/CCM.0000000000003514 30407951

[3] Hurst AL, Child J, Pearce K et al Handshake stewardship: a highly effective rounding-based antimicrobial optimization service. Pediatr Infect Dis J 2016; 35: 1104–10. 10.1097/INF.0000000000001245 27254036

[4] Devchand M, Stewardson AJ, Urbancic KF et al Outcomes of an electronic medical record (EMR)–driven intensive care unit (ICU)-antimicrobial stewardship (AMS) ward round: assessing the “five moments of antimicrobial prescribing”. Infect Control Hosp Epidemiol 2019; 40: 1170–5. 10.1017/ice.2019.218 31407651

[5] Devchand M, Nolen A, Stewardson AJ et al Long-term outcomes of an electronic medical record (EMR)–integrated antimicrobial stewardship (AMS) intensive care unit (ICU) ward round. Infect Control Hosp Epidemiol 2022; 43: 670–2. 10.1017/ice.2021.71 33731236

[6] NAUSP . National Antimicrobial Utilisation Surveillance Program (NAUSP). Government of South Australia; 2025. https://www.sahealth.sa.gov.au/wps/wcm/connect/public+content/sa+health+internet/clinical+resources/clinical+programs+and+practice+guidelines/infection+and+injury+management/antimicrobial+stewardship/national+antimicrobial+utilisation+surveillance+program+nausp/national+antimicrobial+utilisation+surveillance+program+nausp

[7] Chiotos K, Tamma PD, Gerber JS. Antibiotic stewardship in the intensive care unit: challenges and opportunities. Infect Control Hosp Epidemiol 2019; 40: 693–8. 10.1017/ice.2019.7431046851

[8] Hurst AL, Child J, Parker SK. Intervention and acceptance rates support handshake-stewardship strategy. J Pediatric Infect Dis Soc 2019; 8: 162–5. 10.1093/jpids/piy054 29912364

[9] Kit-Anan W, Boonsathorn S, Anantasit N et al Handshake stewardship reduces carbapenem prescription in a pediatric critical care setting. Pediatr Int 2022; 64: e15227. 10.1111/ped.15227 35912458

[10] Shamas N, Stokle E, Ashiru-Oredope D et al Challenges of implementing antimicrobial stewardship tools in low to middle income countries (LMICs). Infect Prev Pract 2023; 5: 100315. 10.1016/j.infpip.2023.100315 38107237 PMC10724472

[11] Durand C, Risso K, Loschi M et al Efficacy of an antimicrobial stewardship intervention for early adaptation of antibiotic therapy in high-risk neutropenic patients. Antimicrob Resist Infect Control 2024; 13: 5. 10.1186/s13756-023-01354-5 38233960 PMC10795280

[12] Contejean A, Abbara S, Chentouh R et al Antimicrobial stewardship in high-risk febrile neutropenia patients. Antimicrob Resist Infect Control 2022; 11: 52. 10.1186/s13756-022-01084-0 35346373 PMC8961889

[13] Charani E, Tarrant C, Moorthy K et al Understanding antibiotic decision making in surgery—a qualitative analysis. Clin Microbiol Infect 2017; 23: 752–60. 10.1016/j.cmi.2017.03.013 28341492

[14] Langford BJ, Nisenbaum R, Brown KA et al Antibiotics: easier to start than to stop? Predictors of antimicrobial stewardship recommendation acceptance. Clin Microbiol Infect 2020; 26: 1638–43. 10.1016/j.cmi.2020.07.048 32771646

[15] Australian Commission on Safety and Quality in Health Care. Surgical National Antimicrobial Prescribing Survey (SNAPS): results of the 2016 pilot. 2016. https://www.safetyandquality.gov.au/sites/default/files/migrated/Surgical-National-Antimicrobial-Prescribing-Survey-SNAPS-Report-Results-of-the-2016-Pilot-November-2017.pdf

[16] Tischendorf J, Giuffre A, Cinnamon K et al Bias and discrimination perceived by antimicrobial stewards: a mixed-methods study. Infect Control Hosp Epidemiol 2025; 46: 910–9. 10.1017/ice.2025.10224 40826921 PMC12616225

[17] Perera D, Vogrin S, Khumra S et al Impact of a sustained, collaborative antimicrobial stewardship programme in spinal cord injury patients. JAC Antimicrob Resist 2023; 5: dlad111. 10.1093/jacamr/dlad111 38021039 PMC10664407

[18] Rose MT, Holmes NE, Eastwood GM et al Oral challenge vs routine care to assess low-risk penicillin allergy in critically ill hospital patients (ORACLE): a pilot safety and feasibility randomised controlled trial. Intensive Care Med 2024; 50: 913–21. 10.1007/s00134-024-07448-x 38739277 PMC11164790

[19] Pandolfo AM, Horne R, Jani Y et al Understanding decisions about antibiotic prescribing in ICU: an application of the necessity concerns framework. BMJ Qual Saf 2022; 31: 199–210. 10.1136/bmjqs-2020-012479 PMC8899486 34099497

[20] Baur D, Gladstone BP, Burkert F et al Effect of antibiotic stewardship on the incidence of infection and colonisation with antibiotic-resistant bacteria and *Clostridium difficile* infection: a systematic review and meta-analysis. Lancet Infect Dis 2017; 17: 990–1001. 10.1016/S1473-3099(17)30325-0 28629876

