# Supplementary material for: Sustaining stewardship: longitudinal evaluation of an integrated antimicrobial programme in the ICU
Source: J Antimicrob Chemother. 2026 Mar 17;81(4):dkag086. doi: 10.1093/jac/dkag086 (PMC13017873; doi:10.1093/jac/dkag086)
Supplement: dkag086_Supplementary_Data [file dkag086_supplementary_data.docx]

**Supplementary Figures & Tables**

**Table S1.** Institutional standard dosing regimens for target antimicrobials and protocol changes during the study period.

Standard adult ICU dosing regimens used at our institution for the target antimicrobials – does not include dosing alterations for renal impairment. A protocol change to 6-hourly dosing for amoxicillin-clavulanate and piperacillin-tazobactam occurred in the late study period; no other major institutional dosing changes were implemented.

| **Antibiotic** | **Standard regime for adults in ICU** |
| --- | --- |
| Ceftriaxone | 2g IV 24-hourly, OR 1g IV 12-hourly |
| Ciprofloxacin | 750mg PO 12-hourly, OR 400mg IV 12-hourly |
| Meropenem | 1g IV 8-hourly |
| Moxifloxacin | 400mg PO/IV 24-hourly |
| Piperacillin and beta lactamase inhibitor | 4.5g IV 6-hourly |
| Vancomycin | Loading dose 25-30mg/kg, then 15-20mg/kg IV 8 to 12-hourly |
| Amoxicillin and beta lactamase inhibitor | 875/125mg PO 1-hourly, OR 1.2g IV 6-hourly |

**Table S2.** Baseline demographics of ICU patients with AMS recommendations

| **Patients** | n = 4610 |
| --- | --- |
| **Age**, median (IQR) | 62 (50 - 73) |
| **Sex**, n (%)  *Male*  *Female* | 2925 (63.4)  1685 (36.6) |
| **Admitting unit**, n (%)  *Medical*  *Surgical* | 2925 (63.4)  1685 (36.6) |
| **Immunocompromised Admitting Units**, n(%)  *Liver Transplant*  *Haematology/Oncology*  *Other** | 835 (18.1)  404 (8.8)  275 (6)  156 (3.3) |

*Includes rheumatology, renal transplant

**Table S3** Change over time in antibiotic use for commonly targeted antibiotics, based on DDD/1000 OBD/month. Note. DDD, defined daily doses; OBD, occupied bed days

| Antibiotic | Pre intervention – change over time (slope) | | At the time of intervention start (Aug 2017) | | Post intervention – change over time | | Slope post vs slope pre |
| --- | --- | --- | --- | --- | --- | --- | --- |
|  | Coefficient  (95% CI) | P | Coefficient  (95% CI) | P | Coefficient  (95% CI) | P | P |
| Ceftriaxone | **-7.92 (-11.8, -4.08)** | **<0.001** | -0.41 (-27.3, 26.4) | 0.976 | 0.27 (-0.17, 0.71) | 0.226 | <0.001 |
| Ciprofloxacin | -2.26 (-6.22, 1.70) | 0.261 | 9.35 (-14.6, 33.3) | 0.440 | **-0.35 (-0.58, -0.11)** | **0.004** | 0.337 |
| Meropenem | 8.76 (-0.68, 18.2) | 0.068 | **-37.7 (-66.1, -9.4)** | **0.010** | **-0.34 (-0.64, -0.05)** | **0.021** | 0.059 |
| Moxifloxacin | 0.62 (-0.19, 1.42) | 0.134 | -2.16 (-6.32, 2.00) | 0.305 | 0.06 (-0.02, 0.13) | 0.124 | 0.177 |
| Piperacillin and beta lactamase inhibitor | **3.62 (1.30, 5.93)** | **0.003** | **-38.9 (-60.4, -17.4)** | **0.001** | 0.32 (-0.01, 0.66) | 0.059 | 0.006 |
| Vancomycin | 11.9 (-2.25, 26.0) | 0.098 | **-111.7 (-193.2, -30.2)** | **0.008** | -0.16 (-0.44, 0.13) | 0.277 | 0.094 |
| Amoxicillin and beta lactamase inhibitor | 1.04 (-1.50, 3.58) | 0.419 | **26.5 (6.92, 46.0)** | **0.009** | **1.23 (0.91, 1.55)** | **<0.001** | 0.882 |

**Table S4** Factors associated with acceptance (univariable)

|  | Overall | | Escalation | | De-escalation | | Discontinuation | | Switch | | Optimisation | |
| --- | --- | --- | --- | --- | --- | --- | --- | --- | --- | --- | --- | --- |
|  | OR (95% CI) | p | OR (95% CI) | p | OR (95% CI) | p | OR (95% CI) | p | OR (95% CI) | p | OR (95% CI) | p |
| Age | 1.00 (0.99, 1.00) | 0.06 | 1.00 (0.98, 1.02) | 0.852 | 1.00 (0.99, 1.01) | 0.822 | 0.99 (0.99, 1.00) | 0.067 | 1.00 (0.99, 1.01) | 0.796 | 0.99 (0.99, 1.00) | 0.194 |
| Age >60 years | 0.88 (0.77, 1.01) | 0.076 | 1.31 (0.69, 2.50) | 0.414 | 0.94 (0.69, 1.29) | 0.713 | 0.82 (0.68, 1.00) | 0.05 | 0.92 (0.60, 1.40) | 0.692 | 0.90 (0.68, 1.18) | 0.445 |
| ICH | 1.31 (1.09, 1.57) | 0.003 | 0.95 (0.43, 2.08) | 0.896 | 1.20 (0.79, 1.82) | 0.393 | 1.33 (1.04, 1.70) | 0.024 | 1.38 (0.80, 2.39) | 0.249 | 1.20 (0.85, 1.68) | 0.298 |
| Medical vs surgical admission | 1.31 (1.14, 1.50) | <0.001 | 0.59 (0.30, 1.19) | 0.139 | 1.26 (0.92, 1.71) | 0.147 | 1.58 (1.30, 1.92) | <0.001 | 1.34 (0.87, 2.05) | 0.185 | 1.12 (0.84, 1.48) | 0.455 |
| Patient sex - Male vs female | 1.01 (0.88, 1.16) | 0.917 | 0.62 (0.31, 1.25) | 0.184 | 0.92 (0.66, 1.28) | 0.609 | 0.94 (0.77, 1.14) | 0.539 | 1.39 (0.91, 2.12) | 0.125 | 1.20 (0.91, 1.58) | 0.194 |
| ID clinician gender - people identifying as men vs women | 1.23 (1.07, 1.40) | 0.003 | 1.73 (0.91, 3.29) | 0.096 | 1.46 (1.06, 2.01) | 0.02 | 1.05 (0.87, 1.27) | 0.612 | 1.31 (0.87, 2.00) | 0.2 | 1.30 (0.99, 1.71) | 0.063 |
| ID clinician seniority - number of years since fellowship | 1.00 (1.00, 1.01) | 0.343 | 1.00 (0.97, 1.03) | 0.876 | 1.03 (1.01, 1.05) | 0.01 | 0.99 (0.98, 1.01) | 0.28 | 0.99 (0.97, 1.02) | 0.656 | 1.01 (0.99, 1.03) | 0.309 |
| Interaction between consultant gender and seniority |  | 0.947 |  | 0.337 |  | 0.386 |  | 0.825 |  | 0.022 |  | 0.781 |

**Table S5** Targeted sub-analysis of de-escalation and discontinuation recommendations

|  | Recommendation not followed | Recommendation followed |
| --- | --- | --- |
| Other Antibiotics | 851 (17.4%) | 4042 (82.6%) |
| Meropenem | 48 (12.3%) | 341 (87.7%) |
|  |  | X2 = 6.514, p = 0.009 |

**Figure S1** Ciprofloxacin utilisation by formulation (IV vs oral)


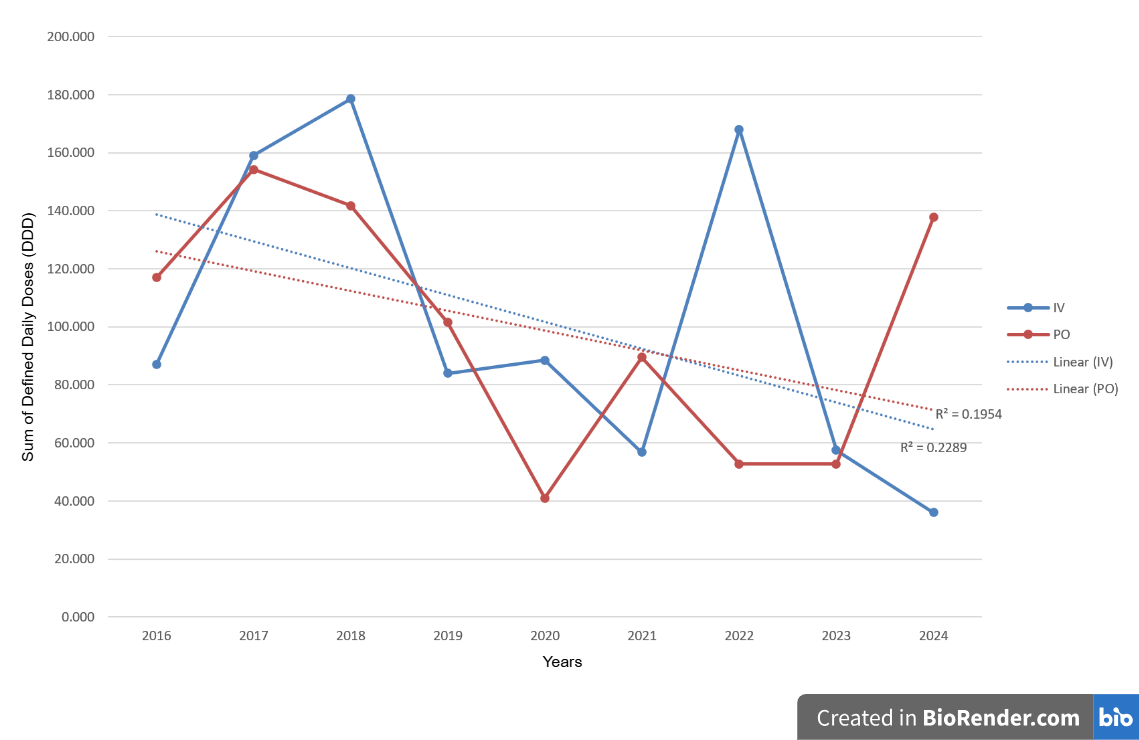


**Figure S2** Amoxicillin-clavulanate utilisation by formulation (IV vs oral)


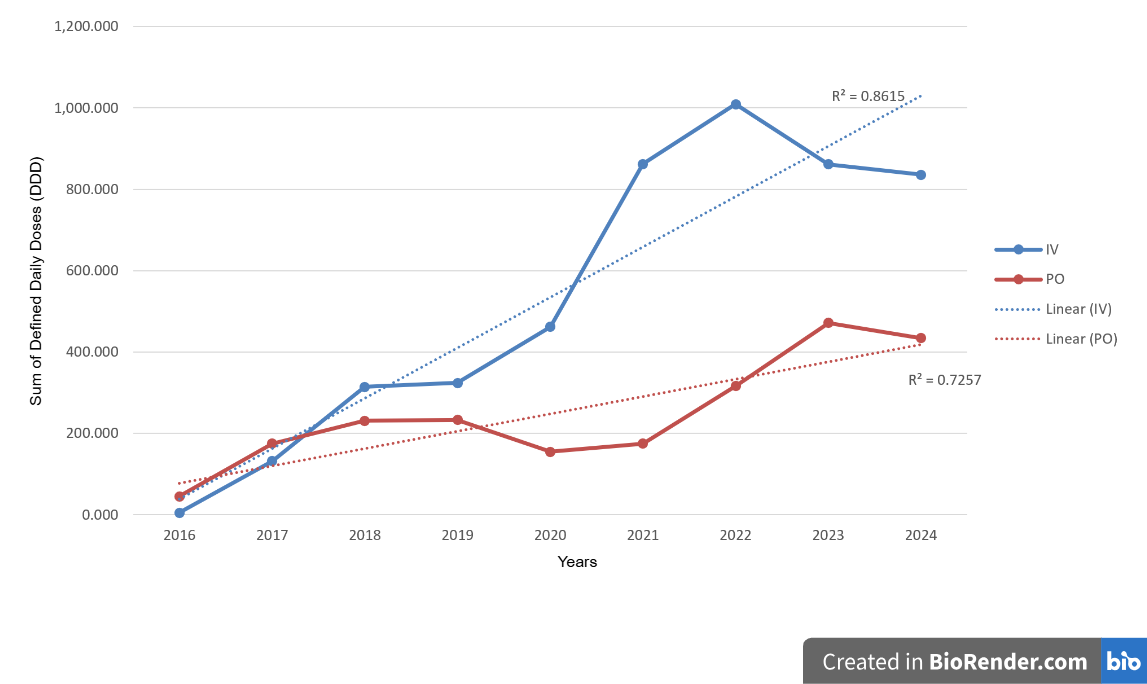


**Figure S3** Recommendations for “5 Moments of Antimicrobial Prescribing” over time ***
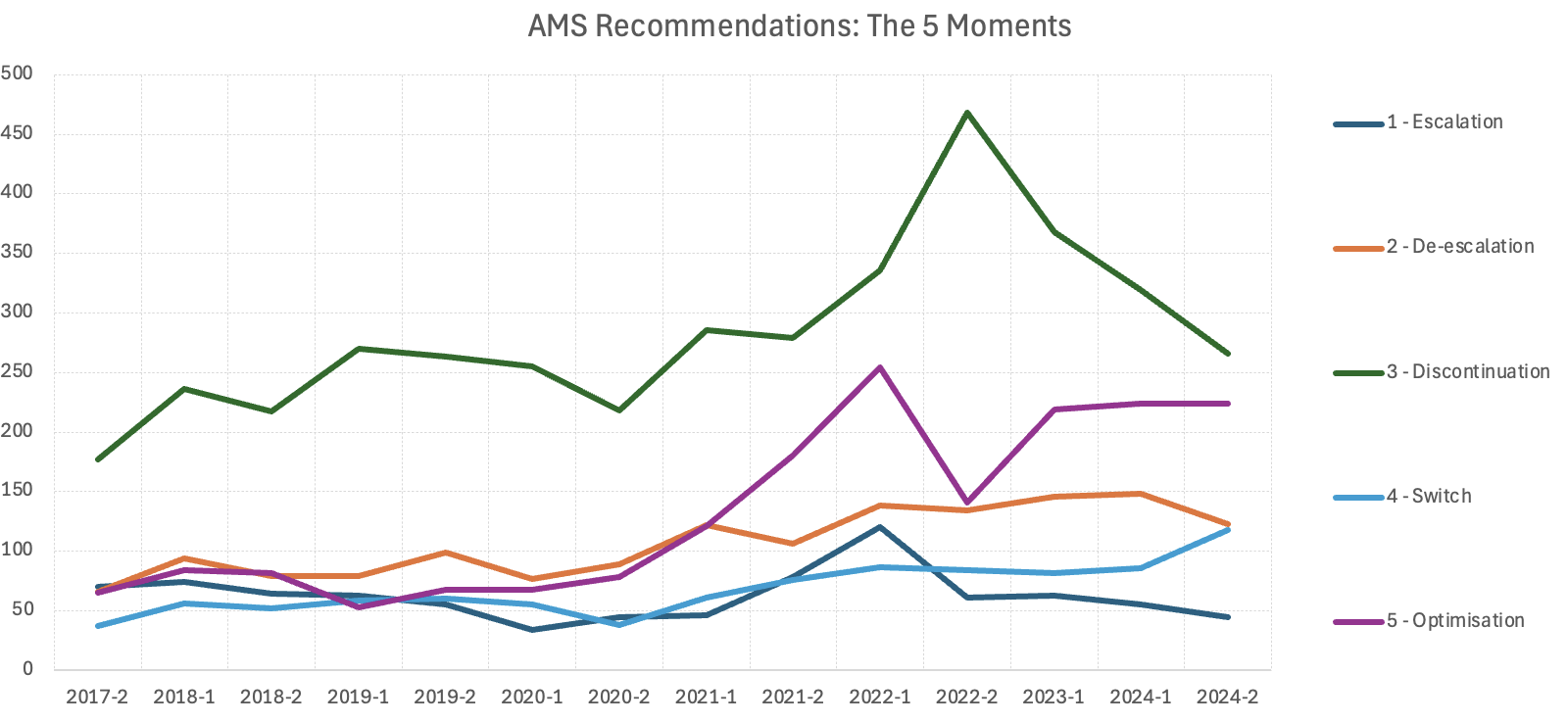
***

**Figure S4** Acceptance rate of AMS recommendations within 24 hours, per “5 Moments of Antimicrobial Prescribing”


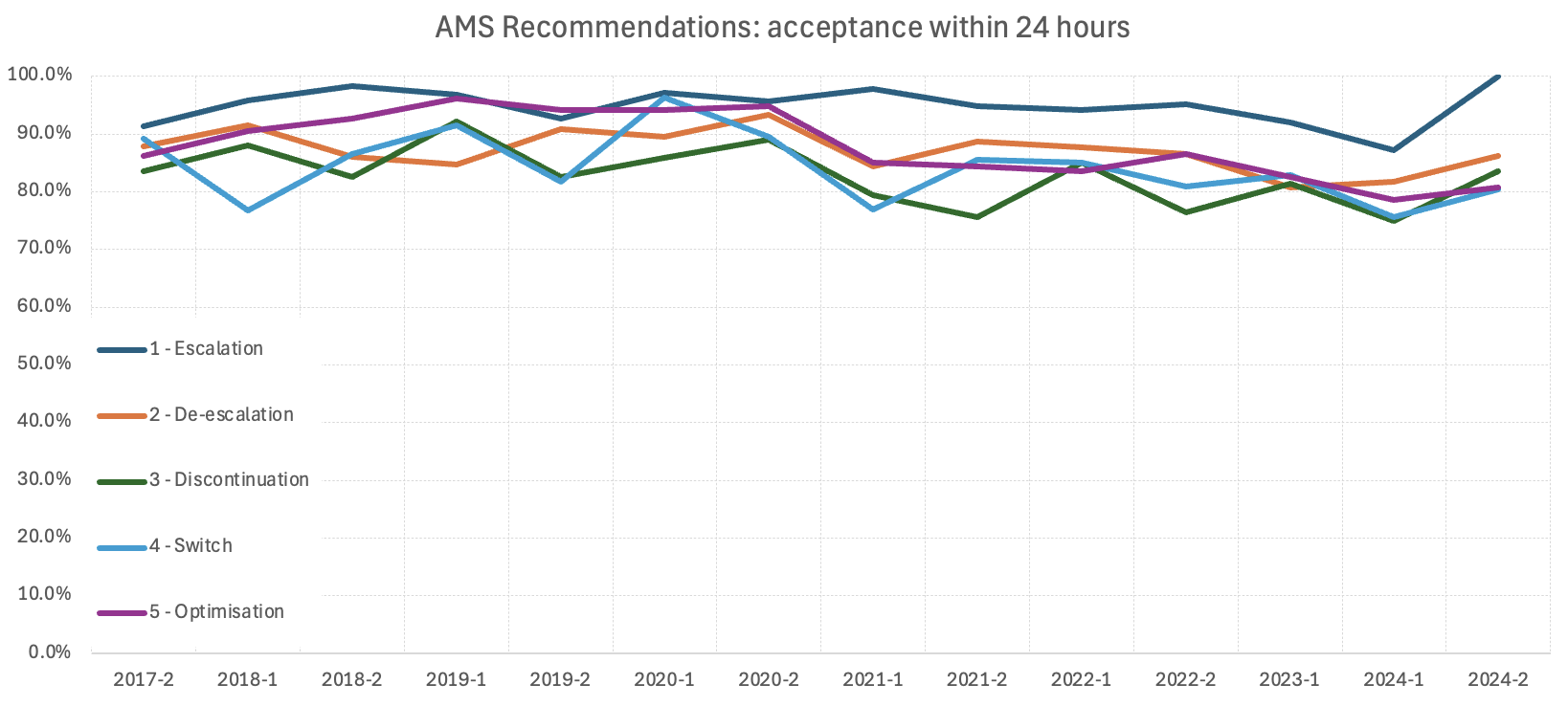


**Figure S5** Australian and New Zealand Risk of Death (ANZROD) Exponentially Weighted Moving Average (EWMA) chart for Austin Hospital – shows sepsis and other infective diagnoses admissions to ICU between July 2017 and June 2024. Report generated on 18 June 2025, from the Adult Patient Database of the Australian and New Zealand Intensive Care Society (ANZICS)


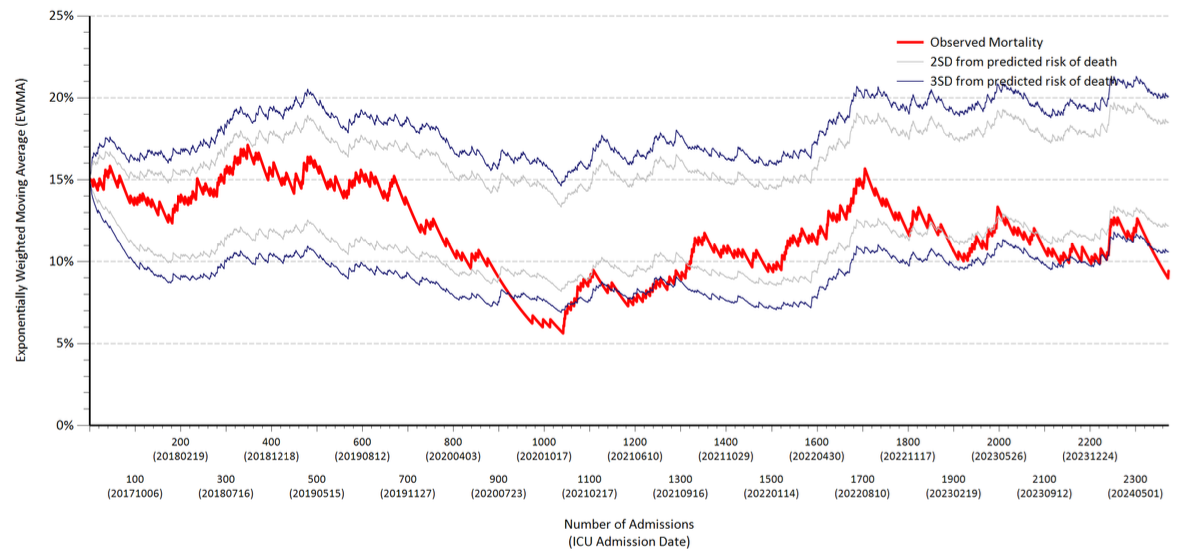


Note: This EWMA chart is based on the ANZROD Model. It shows the weighted moving average of observed in-hospital mortality for patients admitted to ICU and the upper and lower limits of ANZROD predicted mortality. As observed mortality increases the red line moves up; as mortality declines the red line moves down. If patients with greater severity of illness are admitted to the ICU the control lines for predicted mortality (grey and blue) move up. Likewise, if less severely ill patients are admitted to the ICU the predicted mortality control lines move down. Ideally the red line of observed mortality should track within the ANZROD control lines.

Exclusions: Age <16, missing hospital outcome, all physiology missing, patients admitted to ICU for palliative care or organ donation and readmissions to ICU during the same hospital stay.

**Figure S6** Australian and New Zealand Risk of Death (ANZROD) Funnel Plot displaying Standardized Mortality Ratios (SMR) for Austin Hospital (Tertiary, CICM Level 3/PICU) compared with other CICM level 1, 2 and 3/PICU, metropolitan, private, rural / regional, and tertiary hospitals, for ICU admissions with sepsis and other infective diagnoses between July 2017 and June 2024. Report generated on 18 June 2025, from the Adult Patient Database of the Australian and New Zealand Intensive Care Society (ANZICS)


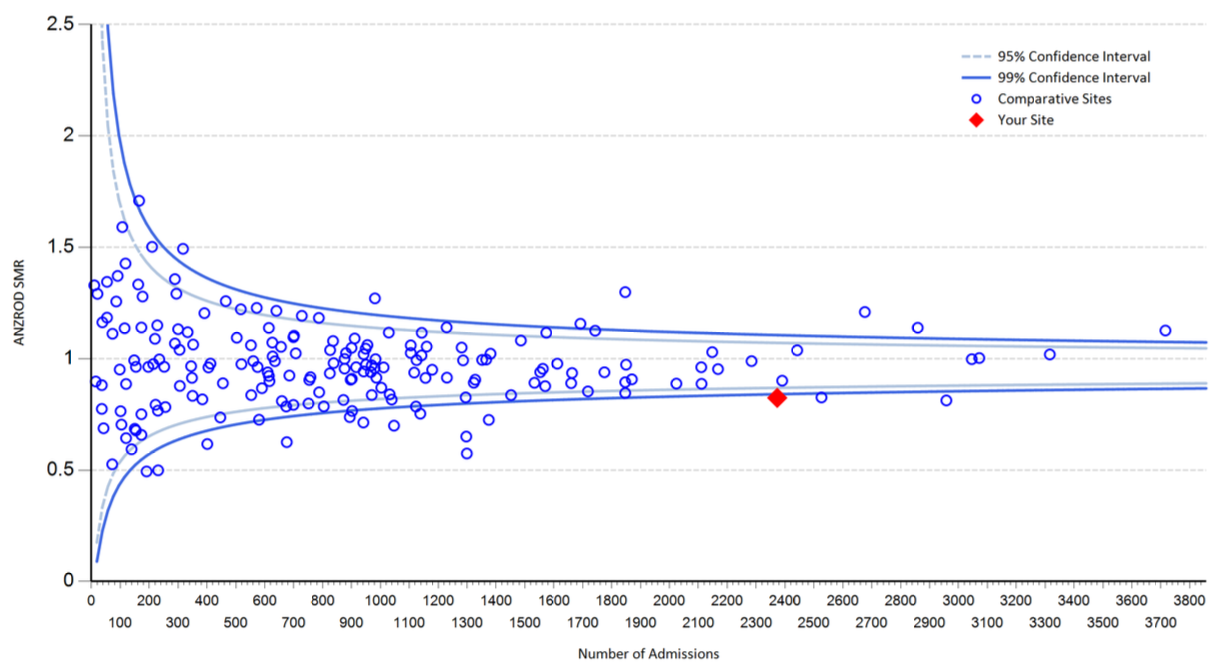


CICM; College of Intensive Care Medicine

PICU; Paediatric Intensive Care Unit

Note: This funnel plot is based on the ANZROD Model. The control lines that form the funnel shape are the 95% and 99% confidence intervals for the mean SMR of the group.

Exclusions: Age <16, missing hospital outcome, all physiology missing, patients admitted to ICU for palliative care or organ donation and readmission to ICU during the same hospital stay.

**Figure S7** Australian and New Zealand Risk of Death (ANZROD) Efficiency Plot displaying standardized mortality ratios vs risk adjusted length of stay for Austin Hospital (Tertiary, CICM Level 3/PICU) compared with other CICM level 1, 2 and 3/PICU, metropolitan, private, rural / regional, and tertiary hospitals, for ICU admissions with sepsis and other infective diagnoses between July 2017 and June 2024. Report generated on 19 June 2025, from the Adult Patient Database of the Australian and New Zealand Intensive Care Society (ANZICS)


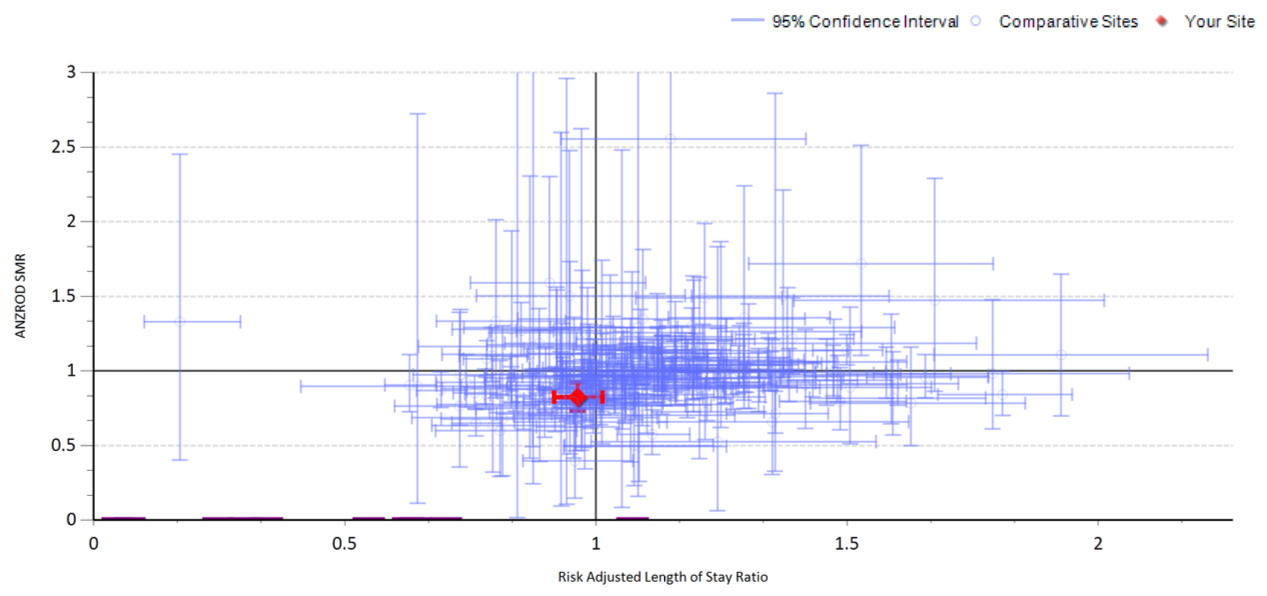


CICM; College of Intensive Care Medicine

PICU; Paediatric Intensive Care Unit

Note: The vertical axis shows the standardised mortality ratio (observed / predicted deaths from the ANZROD Model) with 95% confidence intervals. The horizontal axis shows the risk adjusted ICU length of stay (RALOS) ratio with 95% confidence intervals. This is the ratio of observed length of ICU stay compared to predicted length of. A RALOS of 0.5 indicates the geometric mean of the ICU length of stay for patients at this hospital is half the predicted length of stay.

Exclusions: Age <16, ICU Length of stay >180 days or missing, missing hospital outcome, all physiology missing, patients admitted to ICU for palliative care or organ donation and readmission to ICU during the same hospital stay.
